# The role of dual diagnosis and real-life examples

**DOI:** 10.1192/j.eurpsy.2026.10296

**Published:** 2026-07-31

**Authors:** S. M. M. Toparlak

**Affiliations:** CAMHS, Oxford Health NHS FT, Oxford, United Kingdom

## Abstract

**Abstract:**

Psychiatric rehabilitation remains a cornerstone of comprehensive mental healthcare, yet it continues to be relatively under-emphasised within mainstream service planning and clinical training. While prevention, crisis management, and acute symptom stabilisation are central to psychiatric practice, the longer-term processes that sustain recovery, reduce relapse, and promote meaningful reintegration into society often receive comparatively less attention. Without structured rehabilitation pathways, individuals with severe and enduring mental illness remain at high risk of repeated hospital admissions, social exclusion, unemployment, and functional decline.

This interactive workshop seeks to reposition psychiatric rehabilitation as a critical component of relapse prevention and long-term recovery. It will provide participants with a structured, evidence-based overview of rehabilitation models that aim not only to reduce symptom burden but also to restore autonomy, enhance functional capacity, and support personal recovery goals. Drawing on contemporary research and international service examples, we will examine how comprehensive rehabilitation programmes can improve clinical outcomes, reduce readmission rates, and enhance quality of life.

Through real-life case studies and clinical vignettes, participants will explore the practical application of key interventions, including cognitive behavioural therapy (CBT), life skills training, social skills development, vocational rehabilitation, and supported employment models such as Individual Placement and Support (IPS). We will examine how these interventions contribute to improved community tenure, greater occupational engagement, and strengthened self-efficacy. The session will emphasise the importance of multidisciplinary collaboration between psychiatrists, psychologists, occupational therapists, nurses, social workers, peer support workers, and community agencies. Particular focus will be placed on continuity of care, assertive outreach approaches, and coordinated transitions between inpatient and community services.

The workshop will also explore innovative developments in rehabilitation delivery. Emerging technologies such as mobile health applications for relapse monitoring, digital mood tracking, and telepsychiatry platforms offer promising opportunities to enhance engagement, accessibility, and long-term therapeutic continuity.

Importantly, the session will address gender-sensitive, trauma-informed, and culturally responsive rehabilitation models. Participants will reflect on how rehabilitation frameworks can be adapted to meet the needs of diverse populations, ensuring equitable access and personalised care. Consideration will also be given to recovery-oriented language, co-production with service users, and the integration of lived experience perspectives into service design.

**Disclosure of Interest:**

None Declared

